# Supplementary material for: Cranial ornamentation in the Late Cretaceous nodosaurid ankylosaur Hungarosaurus
Source: PeerJ. 2021 Mar 3;9:e11010. doi: 10.7717/peerj.11010 (PMC7936564; doi:10.7717/peerj.11010)
Supplement: Data S1 [file peerj-09-11010-s001.pdf]

Supplementary data to the manuscript entitled:

Cranial ornamentation in the Late Cretaceous nodosaurid ankylosaur *Hungarosaurus*

Attila Ősi*[1], János Magyar[1], Károly Rosta[2], Matthew Vickaryous[3]

Two of the specimens (MTM PAL 2020.31.1., MTM PAL 2020.32.1.) used in this study have not been osteologically described and taxonomically assigned yet. Here we give a detailed comparative osteological description to support the taxonomic assignment of these specimens.

Description, comparison and taxonomic assignment of the two new skulls

1) MTM PAL 2020.31.1.

This skull (estimated length between 26-32 cm) consists of most of the rostrum including the premaxillae, nasals, the right fragmentary maxilla and the right frontal-supraorbital-?prefrontal-?lacrimal complex (Suppl. Fig. 1). Premaxillae and nasals do not fuse to each other resulting in clearly observable sutural surfaces. Dorsomedial compression and pre-depositional weathering strongly eroded the outer suface of the bones preventing the clear identification of cranial ornamentation. Only the premaxillae show some of the original texture of ornamentation. The inverted U-shaped and large (dorsoventral height ca. 2 cm) premaxillary notch is dorsoventrally as high as mediolaterally wide. All these are diagnostic features of *Hungarosaurus* (Ősi et al., 2019), and are present on MTM PAL 2020.31.1.. Other features, such as the ventrally concave, anterolaterally and laterally rounded cutting margin, the elongate nasal processes (Suppl. Fig. 1C, D), the anteroventrally oblique shelf of the external nares, and the presence of

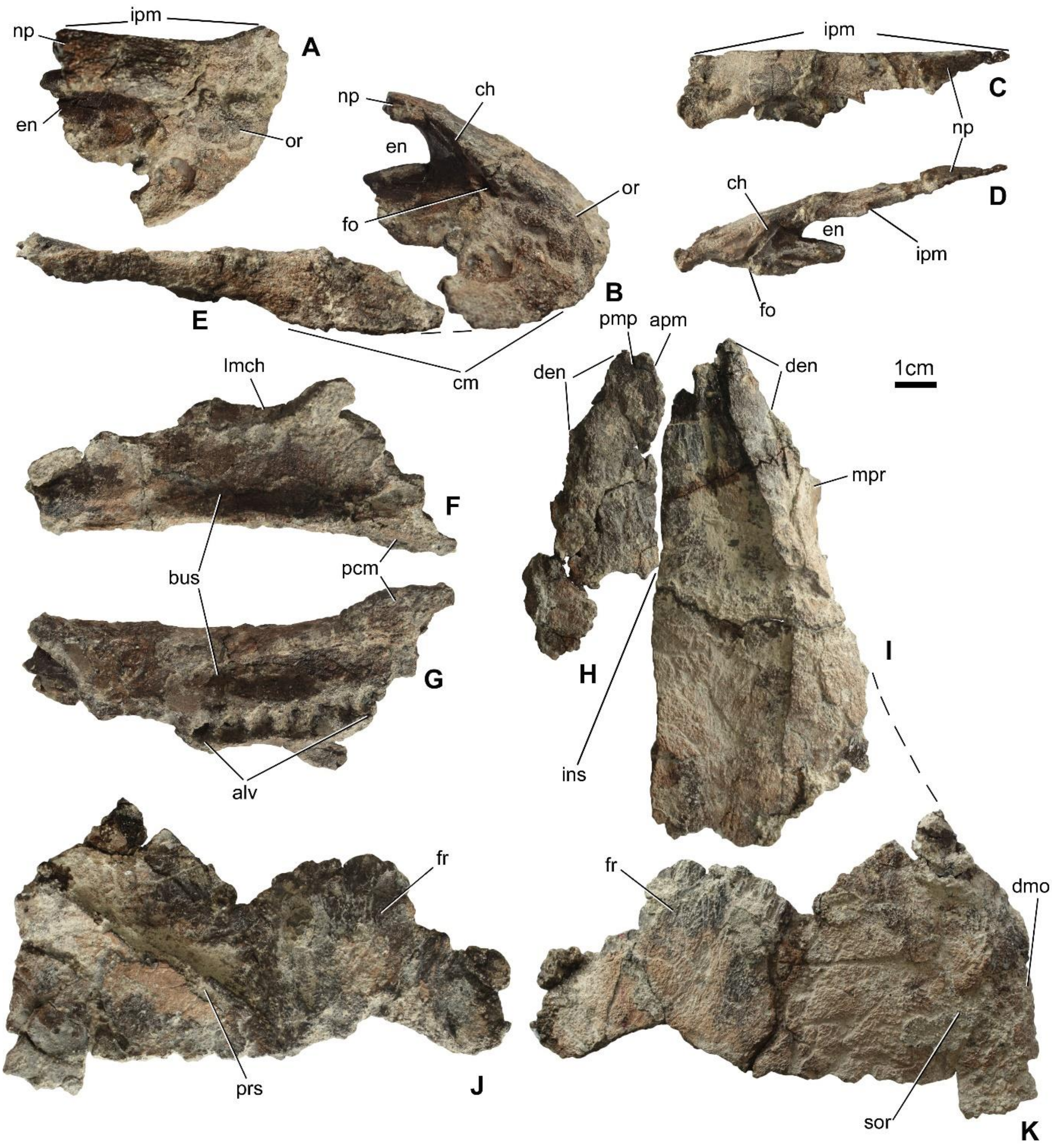

Figure 1. Partial skull MTM PAL 2020.31.1. referred to *Hungarosaurus tormai* from the Santonian of Iharkút, Hungary. A, right premaxilla in dorsal, B, lateral view. C, left premaxilla in dorsal, D, lateral view. E, right maxilla in lateral, F, dorsal, G, ventral view. H, left nasal in dorsal view. I, right nasal in dorsal view. J, interorbital part of the skull roof in ventral, K, dorsal view. Abreviations: alv, alveoli; apm, articular surface for premaxilla; bus, buccal shelf; ch, channel; cm, cutting margin; den, dorsal margin of external nares; dmo, dorsal margin of the orbit; en, external nares; fo, foramen; fr, frontal; ipm, interpremaxillary suture; lmch, lateral margin of choana; mpr, maxillary process of nasal; np, nasal process; or, orbit; pcm, posterior end of cutting margin; pmp, premaxillary process of nasal; prs, preocular shelf; sor, supraorbital.

premaxillary alveoli are also featues shared with *Hungarosaurus*. The posterior end of the nasal process is completely preserved on the left premaxilla having a triangular, very pointed tip (Suppl. Fig. 1C, D) that slightly overlaps the anterodorsomedial margin of the left nasal. Close to the basal part of the nasal process both premaxillae have a ventrally, anteroventrally oriented, laterally opened channel that breaks thorough the premaxilla (Suppl. Fig. 1B) and on the ventral side, it opens into a relatively large (3-4 mm in diameter) foramen. This feature cannot be seen on the holotype of *Hungarosaurus*, and as such might be an ontogenetic or sexual difference. However, it seems to be present on an even smaller, isolated premaxilla (MTM V 2003.12., Ősi and Makádi 2009) suggesting that it might have had the function to supply the ontogenetically growing bone.

Of the two nasals, the right one is more complete being more than twice as long as wide. Anteriorly it becomes narrow to connect to the premaxilla, laterally it curves ventrally to connect with the maxilla and posteriorly it is widest with some slightly eroded scarf joints on its medioventral surface (Suppl. Fig. 1H, I). The internasal suture is a straight, slightly waving, dorsoventrally ca. 3 mm thick surface. Its outer surface is strongly weathered. The anterior part of the left nasal is better preserved having a rounded anterolateral margin bordering dorsally the external nares. The smaller specimen (Suppl. Fig. 1H) is almost identical with the left nasal of the holotype of *Hungarosaurus*.

The fragmentary right maxilla has an anteriorly slightly widening morphology (Suppl. Fig. 1F, G). Anterolaterally the ventral side of the maxilla forms the posteriorly widened cutting margin of the premaxilla. Eight alveoli are preserved in the maxilla, but the posterior half of the alveolar row is missing suggesting at least the double of the number of alveoli. This bone is also compressed dorsomedially, but has a significant buccal shelf (1.5-2 cm wide lateromedially) lateral to the alveolar row, as is present in most ankylosaurs (Vickaryous et al.

2004). Mediodorsal to the anterior alveoli, is a 1 cm long process which curves anterodorsally. This may representthe anteriormost margin of the choana.

A large part of the anterior skull roof between the orbits is preserved. The outer surface is strongly weathered (and relatively thin, max. thickness of 4-6mm) and thus not overly informative, but laterally and anterolaterally it preserves the dorsal and anterodorsal margin of the orbit. No crest or other distinctive cranial ornamentation is present on this part of the orbital margin. The posterior section of the orbit, present in the holotype of *Hungarosaurus*, is not preserved for MTM PAL 2020.31.1. (Suppl. Fig. 1K), thus the presence or absence of a postorbital crest is unkown. Ventrally the preocular shelf extends into the anteroventrally bending anterodorsal corner of the orbital rim (Suppl. Fig. 1J). Sutures between the individual cranial elements on this piece of bone cannot be observed, but according to the position of this element most of the right prefrontal-supraorbital-frontal complex is preserved.

Since there are no corresponding elements with any of the cranial material of *Struthiosaurus* spp. (Seeley 1881, Nopcsa 1929, Pereda-Suberbiola and Galton 1994, 2001), the preserved elements of MTM PAL 2020.31.1. only comparisons with those of *Hungarosaurus*. Most of the rostrum of MTM PAL 2020.31.1. is poorly known as well, and only the premaxilla and anterior end of the nasal can be compared with those of the holotype of *Hungarosaurus*. As mentioned earlier, these bones are extremely similar to the bones found in the holotype of *Hungarosaurus* and no different features can be recognized except for the channel dorsoventrally crossing the premaxilla. Based on these features, the most parsimonious scenario is to refer MTM PAL 2020.31.1. to *Hungarosaurus tormai*. *2) MTM PAL 2020.32.1.*

This specimen represents the smallest among the four skulls. It consists of the partial basicranium, most of the skull roof between and posteriorto orbits, the two nasals, the left

postorbital, left squamosal, most of the left quadrate and the distal end of the right quadrate (Suppl. Fig. 2).

In contrast to MTM PAL 2020.31.1., this specimen is better preserved showing many details of the cranial ornamentation on the dorsal elements. The specimen is slightly compressed dorsoventrally thus the basicranium was pushed into braincase. Posterior to the frontal all bones of the skull roof, braincase and orbital region are completely fused and no sutures can be recognized (Suppl. Fig. 2A, B). Although the two nasals are preserved together, the bones are not fused to each other. Neither are they fused to the maxillae and the bones posterior to the nasal, as it can be seen by the overlapping articulation surfaces preserved on the nasals.

The nasals are at least twice as anteroposteriorly long than wide (although anteriorly they are not completely preserved), dorsally highly ornamented (for details see main text), trapezoid elements. At the nasal-frontal contact, the nasals overlap the anterior process of the frontals (Suppl. Fig. 2A, B).Similar sutural contact can be assumed between the nasal and prefrontal, despite the prefrontals not being preserved, as the posterolaterally facing, wedge-like articulation surfaces of the nasals suggest this condition (Suppl. Fig. 2B). An interfrontal suture may be present between the anterior part of the bones

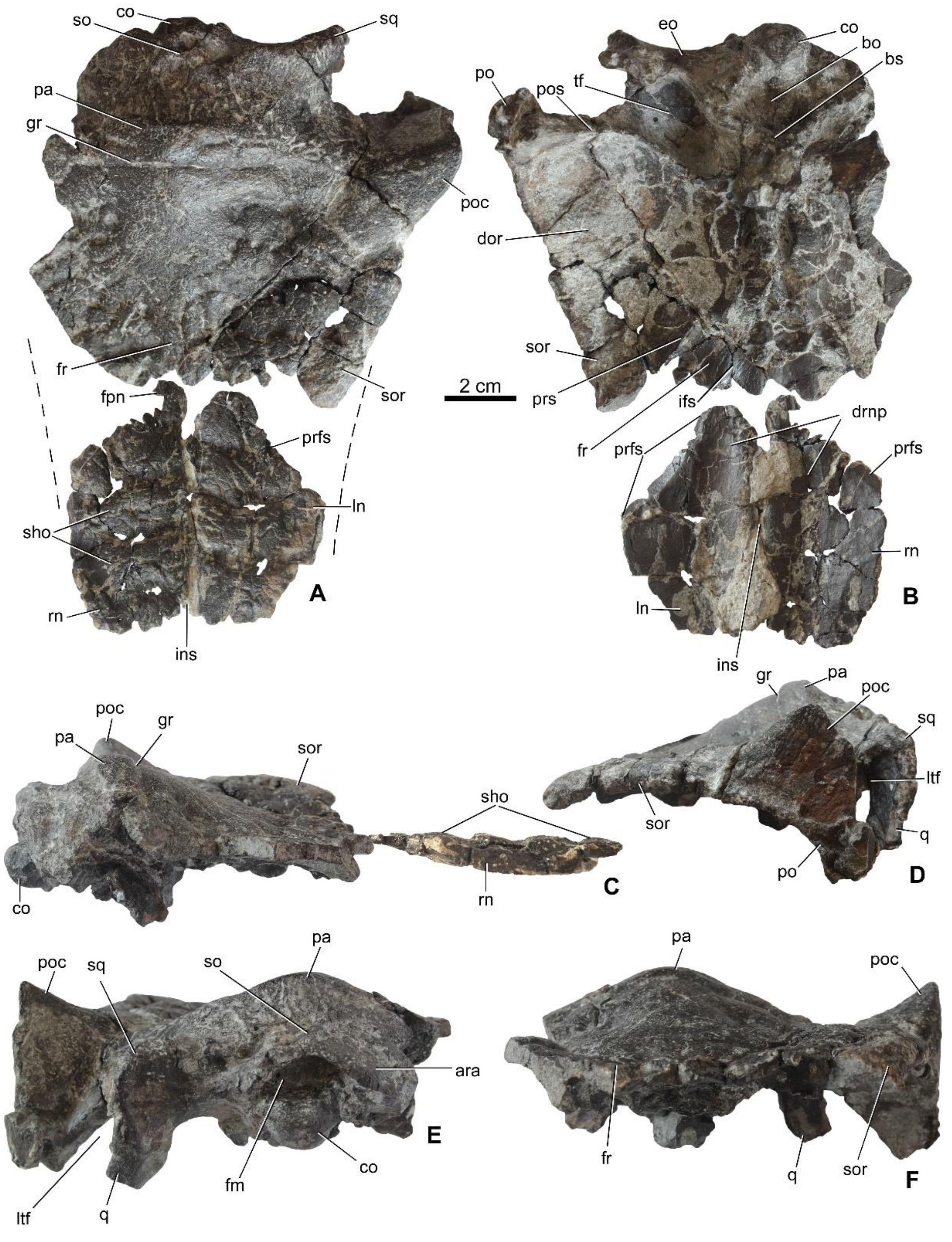

Figure 2. Partial skull MTM PAL 2020.32.1. referred to *Hungarosaurus tormai* from the Santonian of Iharkút, Hungary. A, skull roof and nasals in dorsal, B, ventral, C, right lateral view. D, posterior part of the skull in left lateral, E, posterior, F, anterior view. Abbreviations: ara, articular surface of atlas; bo, basioccipital; bs, basisphenoid; co, condylus occipitalis; dor, dorsal orbital rim; drnp, dorsal rim of narial passage; eo, exoccipital; fm, foramen magnum; fpn, frontal process of nasal; fr, frontal, gr, groove; ins, internasal suture; ln, left nasal; ltf, lateral temporal fenestra; m, maxilla; pa, parietal; po, postorbital; poc, postorbital crest; pos, postocular shelf; prfs, prefrontal suture; prs, preocular shelf; q, quadrate; rn, right nasal; sho, shingled ornamentation; so, supraoccipital; sor, supraorbital; sq, squamosal; tf, temporal fossa.

(also seen weekly on the ventral side), but cranial ornamentation mostly masks it. The anterior part of the nasals are not preserved preventing comparison with that of the holotype of *Hungarosaurus*.

The dorsal surface of the skull roof is highly ornamented. The parietal region is highly vaulted, as seen in *Struthiosaurus* spp., a referred specimen of *Hungarosaurus* (MTM PAL 2013.23.1.) and *Pawpawsaurus* (Lee 1996). The supraoccipital is strongly fused with the parietal and exoccipitals, it bears a short saggital crest. Laterodorsal to the foramen magnum the exoccipital bears an oval, dorsally-posterodorsally facing protuberance, similar to that of MTM PAL 2013.23.1. The condylus occipitalis is wider than high but rather triangular and heart-shaped in posterior-posteroventral view. This is slightly different from the ventrally rounded condyle of the holotype of *Hungarosaurus* and MTM PAL 2013.23.1. It also differs from that of *Struthiosaurus*. The exoccipital-squamosal-quadrate region is a massively fused, L-shaped block. In dorsal view, the squamosal is laterally- posterolaterally oriented and bears no significant ornamentation. Only some rugose texture can be seen on its mediodorsal surface (Suppl. Fig. 2E), extending towards the exoccipital. This may have been served for the attachment of the dorsal neck muscles. The dorsal and posterodorsal part of the left orbital region is preserved in MTM PAL 2020.32.1. Ventrally the orbital fossa is bordered by the posterolaterally extending postocular shelf and anterolaterally by the preocular shelf (Suppl. Fig. 2B). The postorbital bears an anteroposteriorly elongate crest that is relatively shorter and higher than the crest of the holotype of *Hungarosaurus* and MTM 2010.1.1. However, an isolated postorbital crest (MTM 2007.28.1.) from Iharkút shows similar size and morphology. Anterior to the postorbital crest is a slightly ventrally bending dorsally ornamented rim. In lateral view, this margin extends far anteriorly resulting in an abruptly long dorsal margin and would result an enormous orbit. Though the skull was certainly compressed dorsoventrally,

the pre- and postocular shelfs are at a near vertical angle (ca. 85-90°) further suggesting large sized orbits.

One of the quadrates shows the typical L-shape in posterior view separating proximally the lateral temporal fenestra from the posttemporal fenestra (Suppl. Fig. 3B). Mandibular condyle is complete on the right and fragmentary on the left quadrate. The mandibular articulation surface, though much smaller than the type of *Hungarosaurus* or that of *Struthiosaurus* spp. (PIUW 2349, BMNH R 4966), it is more similar to that of *Hungarosaurus* in having a rather rhomboidal than oval shape in distal view (Suppl. Fig. 3C, E). Furthermore, the lateral

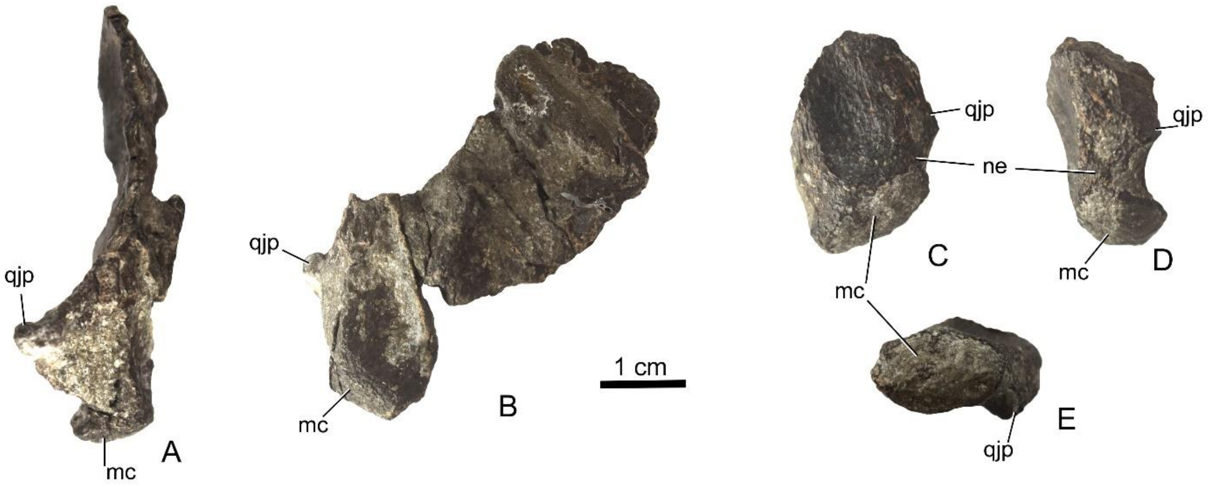

Figure 3. Quadrates of MTM PAL 2020.32.1. referred to *Hungarosaurus tormai* from the Santonian of Iharkút, Hungary. A, left quadrate in lateral, B, posterior view. C, distal part of the right quadrate in posterior, D, lateral, E, distal view.

condyle of the quadrate is separated by a distinct neck from the quadratojugal process (Suppl. Fig. 3C, D) in contrast with the continuous, rounded edge seen in *Struthiosaurus* (BMNH R 4966).

Based on the diagnostic characters of *Hungarosaurus tormai*, such as the postorbital bearing a high and anterodorsal–posteroventrally elongated crest, and the mandibular quadrate condyle having rhomboidal articular surface (Ősi et al. 2019), we herein assign MTM PAL 2020.32.1 to *Hungarosaurus*. However, the very slight differences in the morphology of these characters in conjunction with the relatively large orbits, unfused preorbital bones and the relatively small size of the skull suggests a subadult ontogenetic stage. Thus herein MTM PAL 2020.32.1. is considered as an ontogenetically immature form of *Hungarosaurus tormai*.

References

Lee, Y. N. 1996. A new nodosaurid ankylosaur (Dinosauria: Ornithischia) from the Paw Paw Formation (late Albian) of Texas. Journal of Vertebrate Paleontology, 16(2), 232-245.

Nopcsa, F. 1929. Dinosaurierreste aus Siebenbürgen, V. Geologica Hungarica, series Palaeontologica, 4:1–76.

Ősi, A., & Makádi, L. 2009. New remains of *Hungarosaurus tormai* (Ankylosauria, Dinosauria) from the Upper Cretaceous of Hungary: skeletal reconstruction and body mass estimation. Paläontologische Zeitschrift, 83(2), 227-245.

Ősi, A., Botfalvai, G., Albert, G., & Hajdu, Z. 2019. The dirty dozen: taxonomical and taphonomical overview of a unique ankylosaurian (Dinosauria: Ornithischia) assemblage from the Santonian Iharkút locality, Hungary. Palaeobiodiversity and Palaeoenvironments, 99(2), 195-240.

Pereda Suberbiola, J. and Galton, P.M. 1994. Revision of the cranial features of the dinosaur *Struthiosaurus austriacus* Bunzel (Ornithischia: Ankylosauria) from the Late Cretaceous of Europe. Neues Jahrbuch für Geologie und Paläontologie Abhandlungen,191:173–200.

Pereda Suberbiola, J. and Galton, P.M. 2001. Reappraisal of the nodosaurid ankylosaur *Struthiosaurus austriacus* Bunzel from the Upper Cretaceous Gosau beds of Austria, p. 173–210. In Carpenter, K. (ed.), The Armored Dinosaurs., Indiana University Press, Bloomington.

Seeley, H.G. 1881. On the reptile fauna of the Gosau Formation preserved in the Geological Museum of the University of Vienna. Quarterly Journal of the Geological Society London, 37:620–707.

Vickaryous, M. K., Maryanska, T., & Weishampel, D. B. 2004. Ankylosauria. In Weishampel D. B, Dodson P, & Osmolska H. (Eds.). The Dinosauria (Second Edition). University of California Press, Berkeley, pp. 363-392.
